# Does prehabilitation modify muscle mass in patients with rectal cancer undergoing neoadjuvant therapy? A subanalysis from the REx randomised controlled trial

**DOI:** 10.1007/s10151-020-02262-1

**Published:** 2020-06-20

**Authors:** S. J. Moug, S. J. E. Barry, S. Maguire, N. Johns, D. Dolan, R. J. C. Steele, C. Buchan, G. Mackay, A. S. Anderson, N. Mutrie

**Affiliations:** 1 Department of Surgery, Royal Alexandra Hospital, Corsebar Road, Paisley, PA2 9PN UK; 2 Department of Mathematics and Statistics, Strathclyde University, Glasgow, UK; 3 University of Glasgow, Glasgow, UK; 4 Western General Hospital, Crewe Road South, Edinburgh, UK; 5 Ninewells Hospital and Medical School, Dundee, UK; 6 Department of Surgery, Royal Alexandra Hospital, Paisley, UK; 7 Department of Surgery, Glasgow Royal Infirmary, Glasgow, UK; 8 Division of Cancer Research, Ninewells Medical School, Dundee, UK; 9 Institute of Sport, Physical Education and Health Sciences, Moray House School of Education, Edinburgh, UK

**Keywords:** Prehabilitation, Muscle wasting, Rectal cancer, Sarcopenia, Neoadjuvant chemoradiotherapy, Sarcopenia, Neoadjuvant therapy, Rectal neoplasms, Preoperative care, Walking

## Abstract

**Background:**

Patients with rectal cancer who present with sarcopenia (low muscle mass) are at significantly greater risk of postoperative complications and reduction in disease-free survival. We performed a subanalysis of a randomised controlled study [the REx trial; www.isrctn.com; 62859294] to assess the potential of prehabilitation to modify muscle mass in patients having neoadjuvant chemoradiotherapy (NACRT).

**Methods:**

Patients scheduled for NACRT, then potentially curative surgery (August 2014–March 2016) had baseline physical assessment and psoas muscle mass measurement (total psoas index using computed tomography-based measurements). Participants were randomised to either the intervention (13–17-week telephone-guided graduated walking programme) or control group (standard care). Follow-up testing was performed 1–2 weeks before surgery.

**Results:**

The 44 patients had a mean age of 66.8 years (SD 9.6) and were male (64%); white (98%); American Society of Anesthesiologists class 2 (66%); co-morbid (58%); overweight (72%) (body mass index ≥ 25 kg/m^2^). At baseline, 14% were sarcopenic. At follow-up, 13 (65%) of patients in the prehabilitation group had increased muscle mass versus 7 (35%) that experienced a decrease. Conversely, 16 (67%) controls experienced a decrease in muscle mass and 8 (33%) showed an increase. An adjusted linear regression model estimated a mean treatment difference in Total Psoas Index of 40.2mm^2^/m^2^ (95% CI − 3.4 to 83.7) between groups in change from baseline (*p* = 0.07).

**Conclusions:**

Prehabilitation improved muscle mass in patients with rectal cancer who had NACRT. These results need to be explored in a larger trial to determine if the poorer short- and long-term patient outcomes associated with low muscle mass can be minimised by prehabilitation.

## Introduction

Low muscle mass, or sarcopenia, has been reported in up to 60% of patients with colorectal cancer either at the time of diagnosis, or as a result of neoadjuvant chemoradiotherapy (NACRT) [1–4]. If present, the patient is at higher risk of worse outcomes following surgical resection, including major complications, increased hospital stay and early postoperative mortality [5, 6]. Sarcopenia also appears to influence a patient’s long-term outcome with reduced 1- and 5-year survival in patients with colorectal cancer reported [7, 8]. These poorer outcomes are not restricted to patients with rectal cancer and have been extensively reported in other malignancies including, renal, lung and gastroesophageal [9–12]. However, to date, there are no reported interventions that have modified or offset treatment-related sarcopenia in these cancer populations.

Prehabilitation is an intervention that places emphasis on optimising patients prior to their first treatment and consists predominately of individualised physical activity or exercise programmes. Shown to be safe and feasible in many cancer populations, prehabilitation studies are now reporting on improved patient outcomes after surgery [13–22]. With physical activity and exercise providing physiological overload that increases muscle mass in the general population, prehabilitation in patients with rectal cancer provides an opportunity to determine if low muscle mass can be modified.

Within a study that aimed to assess the feasibility of performing a physical activity intervention during NACRT for rectal cancer, we performed a subanalysis to establish the prevalence of sarcopenia and to determine if a physical activity intervention had the potential to modify muscle mass.

## Materials and methods

### Study design and data collection

From August 2014 to March 2016 (20 months), any adult over 18 years with a new diagnosis of rectal cancer where NACRT was planned was considered for inclusion in the REx trial [23]. The trial was approved by the West of Scotland Research Ethics Service (14/WS/0079) and registered with ISRCTN (www.isrctn.com; 62859294; 17th March 2014). This study was funded by the Chief Scientist Office (CZH/4/986; www.cso.scot.nhs.uk).

This was a two-arm randomised controlled feasibility study (RCT) with the full protocol, physical intervention and results published [23]. Briefly, each participant’s demographics were recorded and they completed baseline-testing including daily step count, physical parameters and psychological. Clinico-pathological outcomes were also recorded. Baseline testing was pre-intervention (Test 1 prior to undergoing NACRT) and repeated post-intervention (1–2 weeks pre-surgery, post-prehabilitation, Test 2).

The walking programme started prior to NACRT and was of minimum 13-week duration. It consisted of graduated step count goals using pedometers and was telephone guided supplemented by walking diaries. The target was for the participants to increase their average daily step count by 3000 accumulated above their baseline by week 8 [24–28]. The control group received standard care and told to maintain their normal level of physical activity.

### Body composition measurement

Muscle mass was measured on each patient using a validated technique: the total cross-sectional area of the psoas muscles [total psoas area, (TPA)] [8]. This was measured manually using a free-hand drawing technique on Picture Archiving and Communication System (PACS) at the level of the L3 vertebra on pre-treatment computed tomography (CT) scan. To ensure standardisation, the exact level of measurement was defined as the CT slice in which both transverse processes were maximally in view. The outline of each individual psoas muscle was traced, the area of each calculated, and summated to provide the TPA (mm^2^). The TPA was then standardised for patient height using the formula TPA (mm^2^)/height (m^2^) to provide the total psoas index (TPI) for each patient. TPI was calculated pre-NACRT and post-NACRT within 4 weeks of planned surgery.

The threshold values used for the diagnosis of sarcopenia were: TPI less than 524 mm^2^/m^2^ for males, and 385 mm^2^/m^2^ for females [29]. To ensure the reliability of our technique, 20 scans were randomly selected and measured for sarcopenia by blinded trained investigators to allow calculation of inter- and intra-class correlation coefficients (ICC). The ICC (*r*^2^) values for inter- and intra-class reliability were 0.957 and 0.985, respectively (close to 1 indicates excellent agreement).

### Statistical analysis

The Mann–Whitney test was used to test for a difference in median change from baseline in TPI between study groups, while the chi-squared test was used to compare the equivalent categorical variable defining sarcopenia (level of significance *p* < 0.050). A linear regression model was fitted to the change from baseline in TPI to assess its association with the study group while adjusting for baseline variables. Spearman’s correlation coefficient was calculated to assess the association between change in TPI and step count. No hypothesis tests were carried out for this part of the analysis due to small numbers. All statistical analysis were undertaken using R (version 3.5.0) [30].

## Results

### Baseline participant characteristics

A total of 48 patients (*n* = 24 in each group) were recruited to the study, of which 44 underwent follow-up CT staging (the 4 who did not were all in the intervention group). The 44 patients had a mean age of 66.8 years (SD 9.6). The majority were male (64%), white (98%), American Society of Anesthesiologists (ASA) class 2 (66%) and co-morbid (58%). Most (59%) participants currently or had previously smoked, 86% reported current alcohol consumption and 72% were overweight (body mass index (BMI) ≥ 25 kg/m^2^) with 20% obese (BMI ≥ 30 kg/m^2^).

Overall, 6 (*n* = 14%) of the 44 patients were sarcopenic at baseline: intervention group 4 (20%) versus control group 2 (8%). Median baseline TPI in the intervention group was 583.5mm^2^/m^2^, and in the control 593.3mm^2^/m^2^ (Table 1).

**Table 1:** Comparison of baseline physical measurements of REx trial participants: Intervention group versus control group, for patients with muscle mass measurements

	All (*N* = 44)	Intervention (*N* = 20)	Control (*N* = 24)
Mean TPI mm^2^/m^2^ (SD)	598 (161)	585 (152)	608 (171)
Median TPI mm^2^/m^2^ (IQR)	584 (460–707)	584 (450–650)	593 (460–718)
Sarcopenic			
No	38 (86%)	16 (80%)	22 (92%)
Yes	6 (14%)	4 (20%)	2 (8%)
Median steps per day^a^(IQR)	6.2 (5.1–10.1)	6.1 (4.7–9.2)	6.7 (5.7–10.3)
Sedentary	10 (23%)	6 (30%)	4 (17%)
Slightly active	13 (30%)	5 (25%)	8 (35%)
Moderately active	16 (37%)	7 (35%)	9 (39%)
Very active	4 (9%)	2 (10%)	2 (9%)
Sit-to-stand test, no. completed in 30 s [mean (SD)]	11.5 (2.7)	11.6 (2.4)	11.4 (3.0)
6-Min walking tests, metres [mean (SD)]	441.3 (63.9)	446.9 (61.9)	436.7 (66.4)
% of week spent active [mean (SD)]	6.6 (2.8)	6.5 (2.9)	6.7 (2.9)
% of week spent sedentary [mean (SD)]	75.9 (12.1)	76.9 (6.1)	74.9 (15.6)

*TPI* Total Psoas Index

^a^Expressed as thousands

None of the 44 participants achieved the recommended government activity guidelines at baseline. The mean number of steps per day of all participants was 6248 (range 1151–17,422 steps) leading to 54% classified as sedentary or only slightly active (Table 1).

### Follow-up results: step count

Median walking intervention duration was 14 weeks (IQR 13–17). At follow-up testing, both groups recorded a reduction in daily step count, with the control group showing a larger reduction (difference between groups in change from baseline of 785 [95% CI − 1194, 2765] adjusted for baseline median daily step count, age and sex). A higher percentage of the intervention group achieved step count improvements at 12 weeks (23.5% versus 15.8%) [19].

### Follow-up results: muscle mass measurement

**Fig. 1:**
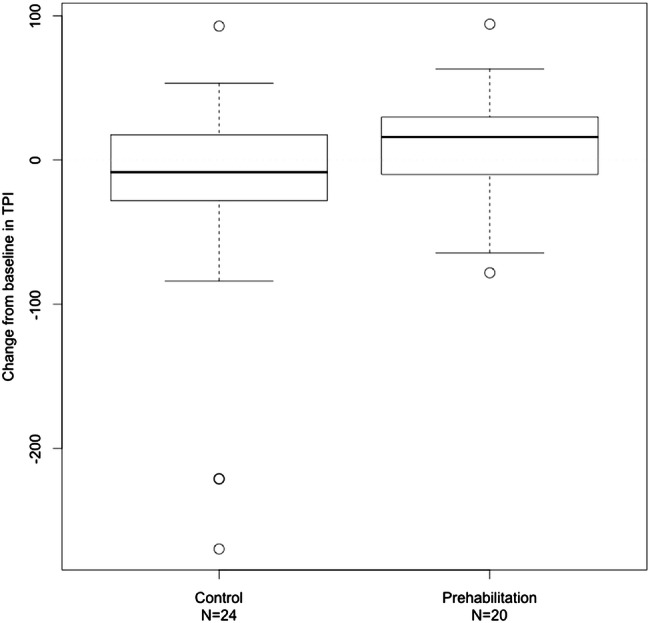
Comparison of change in psoas muscle mass (mm^2^/m^2^) after physical intervention in patients with rectal cancer undergoing chemoradiotherapy. TPI = Total Psoas Index

**Fig. 2:**
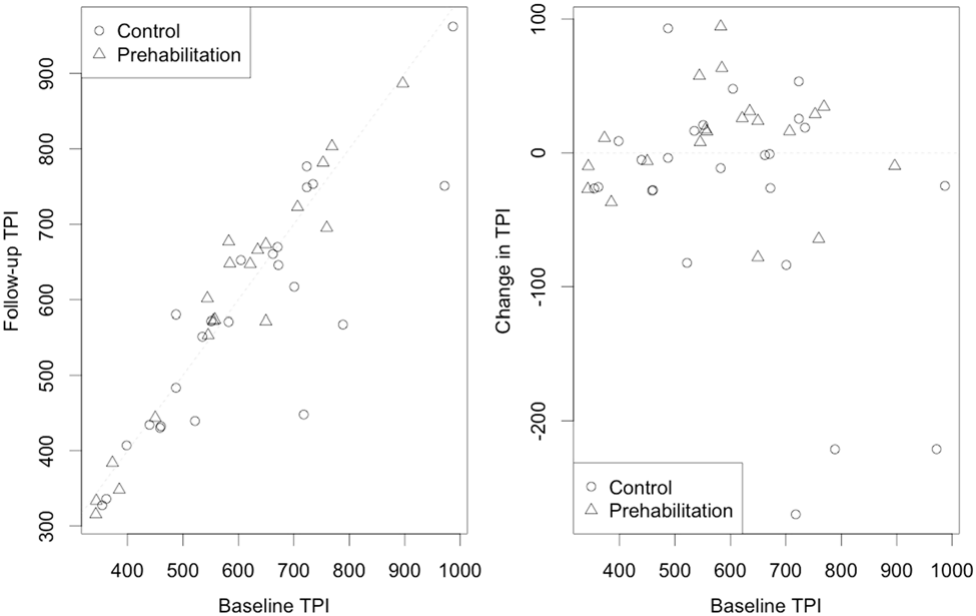
Scatterplots showing Total Psoas Index (TPI) measurements (mm^2^/m^2^) before and after the study period plus change from baseline in patients undergoing neoadjuvant chemoradiotherapy for rectal cancer. The dotted lines represent zero change

The intervention group showed a median increase in TPI of 16.0 mm^2^/m^2^ to a median TPI of 624.2mm^2^/m^2^, compared to a decrease of 8.4mm^2^/m^2^ in the control group to a median TPI of 571.2mm^2^/m^2^ (group difference in median change from baseline of 24.4 mm^2^/m^2^, *p* = 0.07) (Fig. 1). Figure 2 displays the scatter plots for TPI showing that 13 (65%) of patients in the prehabilitation group experienced an increase in muscle mass, versus 7 (35%) that experienced an overall decrease, while 16 (67%) controls experienced a decrease and 8 (33%) showed an increase (*p* = 0.07).

A linear regression model adjusting for age, any comorbidities and baseline TPI estimated a mean treatment difference of 40.2mm^2^/m^2^ (95% CI − 3.4 to 83.7) between the intervention and control groups in change from baseline (*p* = 0.07).

## Discussion

This is the first study to report modification of muscle mass with prehabilitation in patients with colorectal cancer who had neoadjuvant therapy. The graduated individualised walking programme provided sufficient muscle overload to increase psoas muscle mass in 65% of the intervention group in comparison to the controls where 67% had the expected reduction in muscle mass as a consequence of having long-course chemoradiotherapy. With 14% of patients presenting with sarcopenia at diagnosis, prehabilitation may have a further role to play in the perioperative pathway.

Body composition has increasingly been recognised as having an influencing prognostic role in colorectal cancer [5–8, 31]. Despite being modifiable, in contrast to pathological staging, there are few reported interventions to modify muscle mass and potentially improve patient outcomes in the surgical setting [32]. Furthermore, it is not routine practice to screen for low muscle mass and the results from this study show that other physical measurements (daily step count, 6MWT, STS) are not directly related to muscle mass. In particular, BMI is limited as the typical physical appearance of a patient with newly diagnosed colorectal cancer is changing with cancer cachexia becoming increasingly infrequent. This is likely to reflect both the introduction of the NHS Bowel Screening Programme detecting cancers at an earlier stage and of societal behavioural changes in diet and physical activity [33]^.^

Evidence for screening and successful physical activity interventions comes from the gerontology literature where sarcopenia or low muscle mass has been associated with ageing. Prevention or treatment of this muscle mass reduction has been seen as key to enabling older adults to maintain their quality of life and minimise physical limitations [34]. Two recent reviews assessed various forms of exercise in older adults and concluded that resistance training had a lead role in optimising muscle mass and walking capacity [35, 36].

There are several proposed mechanisms for the relationship between low muscle mass and colorectal cancer outcomes. First, it could be that exercise simply provides sufficient physiological overload to improve muscle mass and, therefore, improve mobility as the patient goes through their treatment, minimising cardiorespiratory complications. Step count per se does not appear to relate to psoas muscle mass perhaps because it does not measure intensity and alternative physical capacity measurements could be considered to explore this relationship such as dynamometry. Second, systemic inflammation as a host response to the underlying rectal malignancy has been shown to result in low muscle mass [37]. Third, observational data have consistently shown that patients who are more physically active improve their cancer-specific survival leading to the hypothesis that exercise could have an anti-tumour strategy requiring a precision oncology approach [33, 38]. Finally, the gut microbiome could be a link; alterations in its composition could reduce chronic inflammation and anabolic resistance, leading to an increased muscle mass [39].

Prehabilitation prior to surgery provides an excellent opportunity to modify muscle mass. With an increasing number of publications supporting its feasibility in the colorectal cancer setting, accompanied by increases in aerobic capacity and potentially a reduction in complications, the perioperative care pathway is changing [13–22]. The components of prehabilitation vary between studies with an initial focus on increasing aerobic capacity now expanding to consider psychological and dietary interventions alongside resistance training as stated in the recent Macmillan Prehabilitation Evidence and Insight Review [40]. There is still much work to be done in identifying the optimal prehabilitation pathway for each individual patient, including what components constitute their prehabilitation, what duration and intensity and in what setting: hospital, community and self-led.

There are strengths to this study. We prospectively recruited a population that is often under-reported in research studies, but one that reflects many current surgical practices: older, co-morbid, predominately sedentary, deprived and overweight. With 83% of these participants completing the prehabilitation study, this population should not be excluded from future work. In addition, muscle mass measurement was shown to be a valid, simple and reproducible technique that could be integrated into the NHS with minimal costs.

The authors acknowledge limitations. This was an unplanned subanalysis of a feasibility study so the reader must be cautious about making inferences about the reported outcomes due to very small numbers. In addition, selection bias cannot be excluded with motivated patients potentially more likely to participate and, therefore, achieve better results.

## Conclusions

Patients with rectal cancer presenting with low muscle mass and those developing it as a result of NACRT have poorer short- and long-term patient outcomes as a consequence. This study tentatively reports that a targeted individualised physical activity intervention (prehabilitation) may increase muscle mass and offset the expected reduction with NACRT. This novel work needs to be explored in a larger trial setting to determine the influence of prehabilitation on short- and long-term patient outcomes.
